# A Small KPC-2-Producing Plasmid in *Klebsiella pneumoniae*: Implications for Diversified Vehicles of Carbapenem Resistance

**DOI:** 10.1128/spectrum.02688-21

**Published:** 2022-05-17

**Authors:** Qiwei Chen, Lizhang Liu, Xiaofang Hu, Xu Jia, Xiaowei Gong, Youjun Feng, Man Huang

**Affiliations:** a State Key Laboratory of Veterinary Etiological Biology, Lanzhou Veterinary Research Institute, Chinese Academy of Agricultural Sciences, Lanzhou, Gansu, China; b Department of Microbiology, Zhejiang University School of Medicine, Hangzhou, Zhejiang, China; c General Intensive Care Unit of the Second Affiliated Hospital, Zhejiang University School of Medicine, Hangzhou, Zhejiang, China; d Fuzhou Medical College of Nanchang University, Fuzhou, Jiangxi, China; e Non-coding RNA and Drug Discovery Key Laboratory of Sichuan Province, Chengdu Medical College, Chengdu, Sichuan, China; f College of Animal Sciences, Zhejiang University, Hangzhou, Zhejiang, China; Peking University People’s Hospital

**Keywords:** *Klebsiella pneumoniae*, *K*. *pneumoniae* carbapenemase-2, carbapenem resistance, carbapenem-resistant *K. pneumoniae*, pK186_KPC, small plasmid

## Abstract

The convergence of hypervirulence to carbapenem-resistant K. pneumoniae (CRKP) in a highly transmissible ST11 clone poses a great challenge to public health and anti-infection therapy. Recently, we revealed that an expanding repertoire of diversified KPC-2-producing plasmids occurs in these high-risk clones. Here, we report a clinical case infected with a rare isolate of ST437 CRKP, K186, which exhibited KPC-2 production. Apart from its 5,322,657-bp long chromosome, whole-genome sequencing of strain K186 elucidated three distinct resistance plasmids (designated pK186_1, pK186_2, and pK186_KPC, respectively). Unlike the prevalently larger form of KPC-2-producing plasmids (~120 to ~170 kb) earlier we observed, pK186_KPC is an IncN-type, small plasmid of 26,012bp in length. Combined with the colinear alignment of plasmid genome, the analyses of insertion sequences further suggested that this carbapenem-resistant pK186_KPC might arise from the cointegration of its ancestral IncN and IncFII plasmids, exclusively relying on IS*26*-based transposition events. Taken together, the result represents an unusual example of *bla*_KPC-2_-bearing small plasmids, and highlights an ongoing arsenal of diversified carriers benefiting the transferability of KPC-2 carbapenem resistance.

**IMPORTANCE** A rare ST437 isolate termed K186 was clinically determined which was unlike ST11, the dominant sequence type of CRKP. Whole-genome sequencing enabled us to discover three distinct resistance plasmids, namely, pK186_1, pK186_2, and pK186_KPC. Among them, pK186_KPC appears as a unique plasmid ~26 kb in size, much smaller than the prevalent forms (~120 to ~170 kb). Intriguingly, genetic analysis suggests that it might originate from Proteus mirabilis. This result constitutes an additional example of differentiated plasmid vehicles dedicated to the emergence and dissemination of KPC-2 carbapenem resistance.

## INTRODUCTION

Klebsiella pneumoniae is a major human pathogen because it causes both hospital-acquired infections and community-acquired infections (1–3). Pyogenic liver abscesses caused by K. pneumoniae are becoming a serious problem in clinical settings in Asian countries, of which Taiwan has over 3,000 cases per year (4, 5). Generally, K. pneumoniae of global public health concern includes, but is not limited to, three major subpopulations with distinct phenotypes. Unlike hypervirulent K. pneumoniae (hvKP), with the dominant sequence type ST23 (6), carbapenem-resistant K. pneumoniae (CRKP) is frequently linked to ST11 (3, 7). Worryingly, the third type of K. pneumoniae is hypervirulent CRKP, i.e., hypervirulence and carbapenem resistance converge via a certain plasmid in a single ST11 clone (8). Because pLVPK-like virulence plasmids are generally nonconjugative (9, 10), we speculated that this convergence is due to the acquisition of a carbapenem-resistance plasmid by a virulent, highly transmissible ST11 clone (11). In contrast, Yang et al. (12) reported a rare case of a conjugative virulence plasmid, p15WZ-82_Vir. Genetic analysis hypothesized that the formation of p15WZ-82_Vir is due to the integration of a pLVPK-type virulence plasmid fragment (~100 kb) into a conjugative IncFIB plasmid backbone (12). Thus, the emergence of conjugative virulence plasmids benefits the rapid spread of virulence across Enterobacterales, including K. pneumoniae, producing the so-called ‘superbug’ hypervirulent CRKP. As a result, this pathogen might challenge global public health and anti-infection therapy.

To the best of our knowledge, there are two types of genetic mechanisms accounting for carbapenem resistance, namely, (i) an array of New Delhi metallo-β-lactamase (NDM) variants (NDM-1 to -29) and (ii) a broad range of K. pneumoniae carbapenemase (KPC) subtypes. Of the 88 known KPC-resistance enzymes (KPC-2 to -9, KPC-11 to -82, KPC-84 to -88, KPC-90, KPC-91, and KPC-94 to -95) (13), KPC-2 is the prevalent form in ST11 CRKP. Also, it seems likely that NDM-1 frequently co-transfers with KPC-2 in certain clinical isolates of K. pneumoniae (14). As for human infections with hypervirulent ST11 CRKP in Zhejiang Province, China, Gu et al. (8) reported a KPC-2-resistance plasmid of ~177 kb (pKPC-CR-HvKP4) and a virulence plasmid of ~180 kb (pVir-CR-HvKP4). Additionally, Li et al. (11) identified a different form of *bla*_KPC-2_-bearing IncR plasmids which were ~120 kb in length, instead of ~170 kb. Together with the earlier findings of Li et al. (11), this discovery augments the diversity of KPC-2 transferability. As for most of the K. pneumoniae clinical isolates from this hospital, the virulence plasmid-borne *rmpA2*, which presumably encodes a regulator of mucoid phenotype A, was found to be inactive because of genetic deletion.

Of the six frequently isolated human disease causatives (namely E. coli, Staphylococcus aureus, K. pneumoniae, Enterococcus faecalis, Pseudomonas aeruginosa, and Proteus mirabilis) (15), P. mirabilis results in human urinary tract infections and evolves certain resistance mechanism to individual antibiotics. Retrospectively, the first case of P. mirabilis expressing KPC-2 carbapenem resistance was unveiled in an epidemiological surveillance of blood cultures from a diabetic patient in the US in 2008 (16). The genetic determinant of *bla*_KPC-2_ was soon detected in clinical isolates of P. mirabilis from a certain tertiary hospital in Hangzhou City, China from 2010 to 2012 (17, 18). Plasmid-borne KPC-2 was also clinically tracked in certain multidrug-resistant P. mirabilis in Brazil in 2015 (19). Therefore, it is possible that cross-species transfer of KPC-2 carbapenem resistance between K. pneumoniae and P. mirabilis is mediated by certain plasmids.

Here, we report a follow-up study on the heterogeneity of KPC-2-producing plasmids arising from CRKP isolates from the same hospital in Zhejiang Province. In addition to an unusual virulence plasmid lacking previously known virulence factors, we discovered a small IncN-type, *bla*_KPC-2_-harboring plasmid (termed pK186_KPC, ~26 kb) in ST437 rather than in the transmissible ST11 clone. This is unusual, but not unprecedented. This is because a smaller but highly similar plasmid, pT211 (~24 kb), producing KPC-2, coexists with a larger *bla*_KPC-2_-containing plasmid, pT18, (~59 kb) in a clinical strain of multidrug-resistant P. mirabilis (20). These observations enabled us to formulate a cointegration model for pK186_KPC formation (Fig. 4). This study extends our understanding of diversified vehicles for the emergence and transferability of KPC-2 carbapenem resistance in the clinical sector.

## RESULTS AND DISCUSSION

### Clinical description.

**FIG 1:**
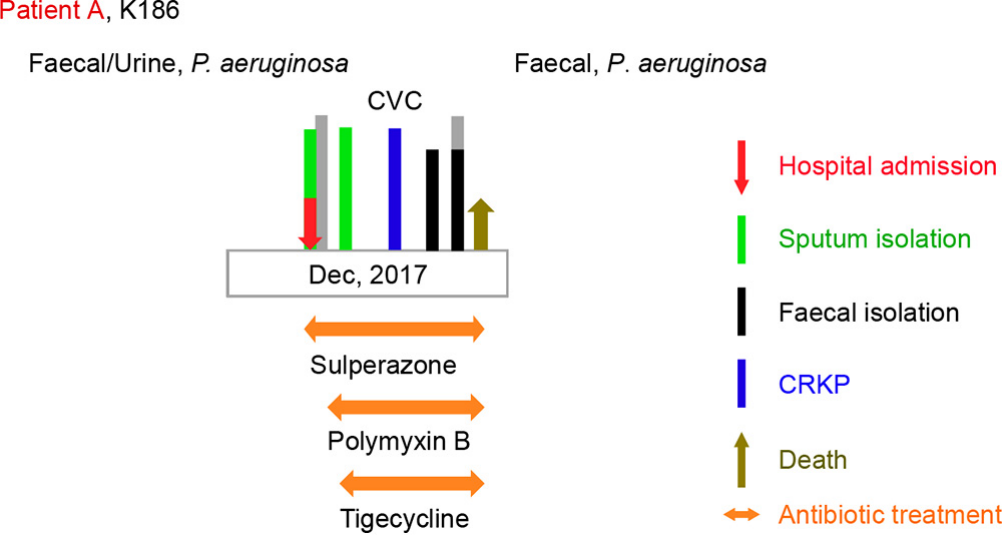
Clinical characterization of patient A, infected with K. pneumoniae K186.

Prior to hospitalization at the Second Affiliated Hospital of Zhejiang University in December 2017, the patient (Patient A) was admitted to the ICU (intensive care unit) at another hospital due to pneumonia 1 month prior (Fig. 1). Computerized tomography images showed a space-occupying lesion in the upper lobe of the right lung, multiple inflammations in both lungs, and a pleural effusion in the left lung. Therefore, Patient A was diagnosed with severe pneumonia and lung cancer.

After admission to the general ICU, Patient A exhibited elevated infection indices, including an elevated white blood cell count, neutrophil ratio, C-reactive protein level, and procalcitonin level. Examination of his sputum culture indicated the presence of carbapenem-resistant K. pneumoniae. It was noted that both the pleural effusion and central venous catheter were confirmed positive for CRKP. A carbapenem-resistant K. pneumoniae strain, termed K186, was isolated from this patient. Subsequently, polymyxin combined with tigecycline was applied to treat this intractable infection. Because his upper limb and face were convulsed during this period, this patient was also diagnosed with secondary epilepsy and received treatment with depakine. Additionally, this patient was treated with intermittent renal replacement therapy (RRT) due to obvious systemic edema with a progressive increase in creatinine (Fig. 1). Unfortunately, treatment with multiple antibiotics (such as imipenem and linezolid) failed to significantly improve Patient A’s condition (Fig. 1).

### Characterization of *K. pneumoniae* K186.

Along with matrix-assisted laser desorption ionization–time of flight mass spectrometry (MALDI-TOF MS) analysis, 16S rRNA gene sequencing confirmed that the isolate K186 was K. pneumoniae. Antibiotic susceptibility assays showed that (i) K186 was resistant to all β-lactam antibiotics tested, such as imipenem and meropenem (MIC ranging from 4 μg/mL to 64 μg/mL); (ii) K186 exhibited resistance to amikacin (MIC, 8 μg/mL), ciprofloxacin (MIC, >2 μg/mL), gentamicin (MIC, >8 μg/mL), and tobramycin (MIC, >8 μg/mL); and (iii) K186 exhibited tigecycline resistance (MIC, 4 μg/mL) (Table 1). To gain genomic insight into this clinical isolate, K186 was subjected to whole-genome sequencing. A total of 91,412 clean reads were harvested with an average length of 15,716 bp (Table S1 in the supplemental material). The resultant four contigs were assembled into a 5,322,657-bp long genome with a GC percentage of 57.5%. In brief, it encodes 5,618 genes, 142 pseudogenes, 25 rRNAs, 85 tRNAs, and 11 noncoding RNAs (accession no. CP076518 to CP076521). Genomic analysis of K186 suggested that its O-locus denotes O4, and its K-locus is KL36. Instead of the dominant ST11 (*gapA*, 3; *infB*, 3; *rpoB*, 1; *mdh*,1; *phoE*, 1; *pgi*, 1; and *tonB*, 1), strain K186 belongs to ST437 (*gapA*, 3; *infB*, 3; *rpoB*, 1; *mdh*, 1; *phoE*, 1; *pgi*, 1; and *tonB*, 31). Clearly, the difference between ST11 and ST437 lies in a single housekeeping gene, *tonB*. As expected, a number of antimicrobial resistance (AMR) genes were detected on its chromosome, including *oqxAB* (quinolone efflux pump), *fosA* (fosfomycin), and *bla*_SHV-182_ (extended-spectrum β-lactamase, ESBL) (Table 2).

**TABLE 1:** Antibiotic resistance profile of K186, clinical isolate of K. pneumoniae

Antibiotic	MIC (μg/mL)	Genetic element	Antimicrobial class
Amikacin	8	*rmtB*	Aminoglycoside
Ciprofloxacin	>2	*oqxAB*	Fluoroquinolone
Gentamicin	>8	*rmtB*	Aminoglycoside
Tobramycin	>8	*rmtB*	Aminoglycoside
Tigecycline	4	NA^*a*^	Tetracycline
Piperacillin	>64	*bla*_KPC-2_	β-lactam
Imipenem	>8	*bla*_KPC-2_	β-lactam
Ertapenem	>4	*bla*_KPC-2_	β-lactam
Cefepime	16	*bla*_KPC-2_	β-lactam
Ceftriaxone	>32	*bla*_KPC-2_	β-lactam
Cefazolin	>32	*bla*_KPC-2_	β-lactam
Aztreonam	>32	*bla*_KPC-2_	β-lactam
Amoxicillin	>16	*bla*_KPC-2_	β-lactam
Meropenem	>4	*bla*_KPC-2_	β-lactam

a NA, not applicable.

**TABLE 2:** Genetic description of genome and plasmids of strain K186^*a*^

Sequence	Plasmid type	Size (bp)	GC (%)	AMR genes	oriTfinder results
Chromosome	NA	5,322,657	57.5	*oqxAB*, *fosA*, *bla*_SHV-182_	NA
pK186_1	IncFII_K_/IncFIB_K_	247,566	51.5	*dfrA12*, *sul3*, *aph(3′)-la*, *aadA1*, *aadA2*, *mef(B)*, *cmlA1*	oriT, relaxase, T4CP, T4SS
pK186_2	IncFII/IncX1	78,128	50.0	*rmtB*	Relaxase, T4CP, T4SS
pK186_KPC	IncN	26,021	54.1	*bla*_KPC-2_	oriT

a NA, not applicable; oriT, origin of tansfer site.

**FIG 2:**
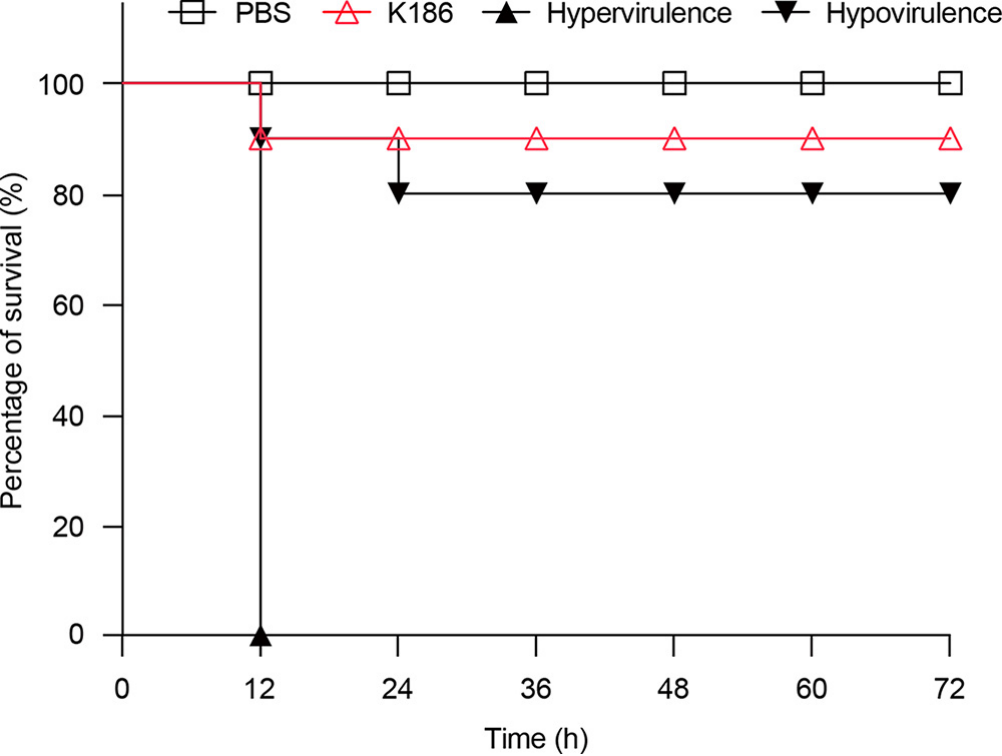
Wax moth larvae-based evidence for the limited virulence of the K. pneumoniae K186 strain. Survival curve is plotted using representative data from three independent infection trials. Galleria mellonella (4 groups, 10 larvae per group) was challenged with log-phase culture K186 at doses of 1 × 10^6^ CFU and recorded for 72 h postinfection. Here, the group inoculated with phosphate-buffered saline (PBS) is used as a blank control, K199 is the hypervirulent strain, and WNX-1 is the hypovirulent strain (11).

Considering that a number of ST11 K. pneumoniae strains arising from this hospital displayed various levels of virulence (11), we were interested in the infectivity of K186, an isolate of ST437 K. pneumoniae. The fact that K186 strain was negative for the string test suggested a lack of high viscosity. It was generally consistent in these scenarios that the K186 strain only demonstrated limited virulence in a wax moth larvae infection model (Fig. 2). The results for the mouse infection model were not completely consistent with the overstatement by Gu et al. (8) that hypervirulent K. pneumoniae with KPC-2 carbapenem resistance is the dominant clone at the Second Affiliated Hospital of Zhejiang University (Hangzhou, China). In contrast, it augments the proposal that a population of diverse K. pneumoniae isolates circulates in certain tertiary hospitals in Zhejiang Province (11).

### Genetic analysis of pK186_KPC.

**FIG 3:**
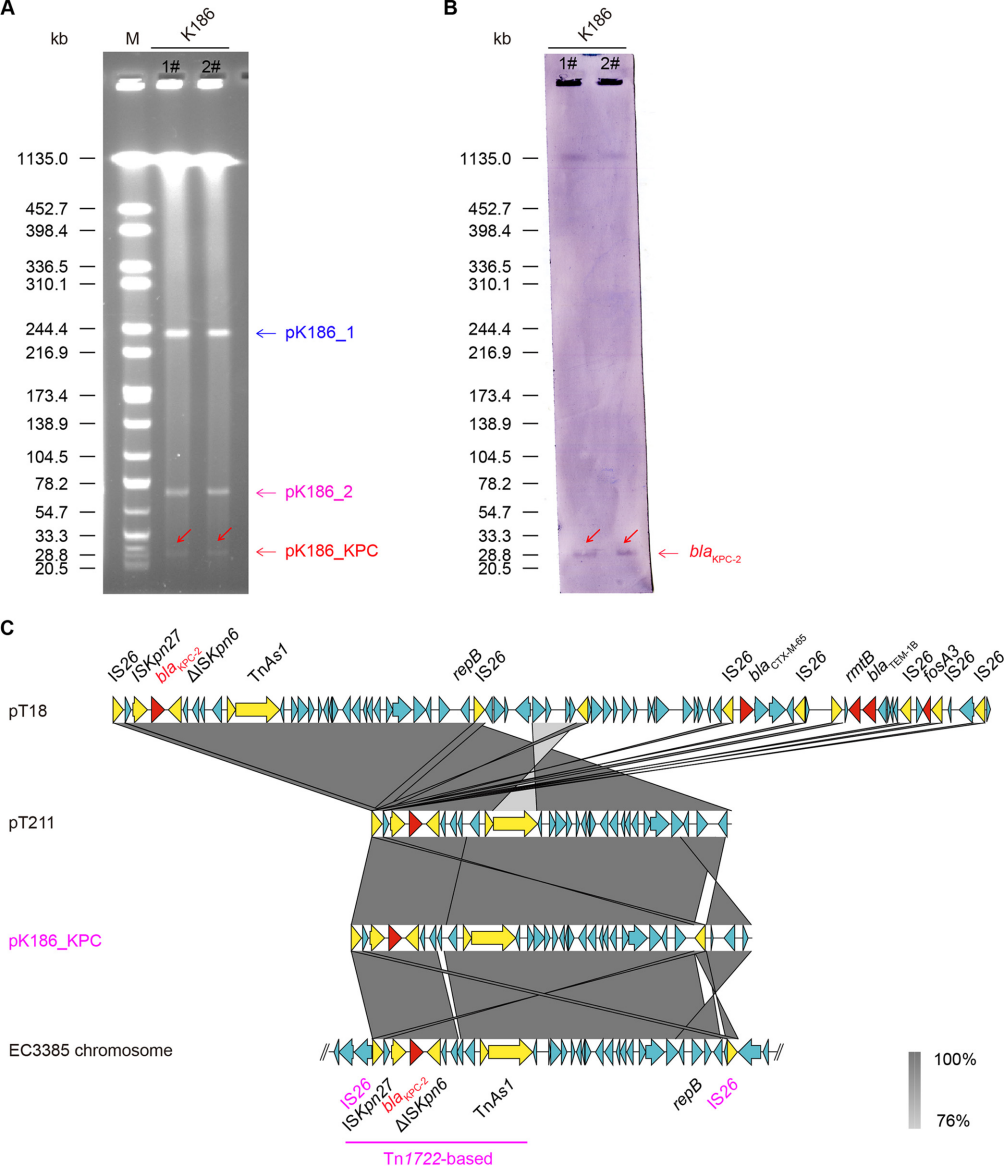
Genetic analysis of the carbapenem-resistant plasmid pK186_KPC. (A) S1-pulsed field gel electrophoresis (S1-PFGE) analysis of strain K186 reveals three distinct plasmids, namely, pK186_1 (~240 kb), pK186_2 (~70 kb), and pK186_KPC. (B) Use of Southern blotting to detect the *bla*_KPC-2_-carrying plasmid. (C) Genomic analyses of the IncN-type plasmid pK186_KPC. In addition to the chromosome sequence of E. coli EC3385 (accession no. CP029420) (41), two additional KPC-2-producing plasmids of Proteus mirabilis are involved (20); namely, pT211 (~24.2 kb; accession no. CP017083) and pT18 (~59 kb; CP017086). Regions of >76% similarity are marked by black shading. The *bla*_KPC-2_ gene is labeled in red, and yellow arrows denote insertion sequences. Presumably, the *bla*_KPC-2_-containing region (~26 kb) is horizontally transferred among the P. mirabilis plasmid, the K. pneumoniae plasmid, and the E. coli chromosome.

In general consistency with the S1-pulsed field gel electrophoresis (S1-PFGE) results (Fig. 3A), whole-genome sequencing revealed that the K186 strain also harbors three additional plasmids, designated pK186_1, pK186_2, and pK186_KPC (Table 2). Among these, pK186_KPC is a *bla*_KPC-2_-bearing plasmid of ~28 kb in length, as verified by Southern blotting with a specific *bla*_KPC-2_ probe (Fig. 3B). Unlike pK187_KPC, a prevalent KPC-2-positive plasmid of roughly 120 kb recently described from the same hospital (11), pK186_KPC is a small KPC-2-producing plasmid. Whole-genome sequencing determined that pK186_KPC is 26,012 bp in length with an average GC percentage of 54.1% (Table 2). Combined with a BLASTn search, PlasmidFinder analysis further determined that pK186_KPC belongs to an IncN-type plasmid consisting of 30 putative open reading frames (Fig. 3B). Linear alignment of plasmid genomes showed that pK186_KPC is well matched with two known plasmids restricted to Proteus mirabilis, namely, pT18 (accession no. CP017086) and pT211 (CP017083). It produced an appreciable level of similarity (100% coverage and 99.96% identity). In addition, pK186_KPC was perfectly aligned with the chromosome sequence of E. coli isolate EC3385 (98% coverage and 99.95% identity) (Fig. 3B). In contrast to the other three references, parts of pK186_KPC were inverted, implying a relic of an IS*26*-mediated inversion event. This was also verified by the observation that two incomplete mobile elements (IS*Kpn27* and ΔIS*Kpn6*) were located directly upstream from *bla*_KPC-2_ in pK186_KPC (Fig. 3B).

### Diversity of KPC-2-producing plasmids.

**FIG 4:**
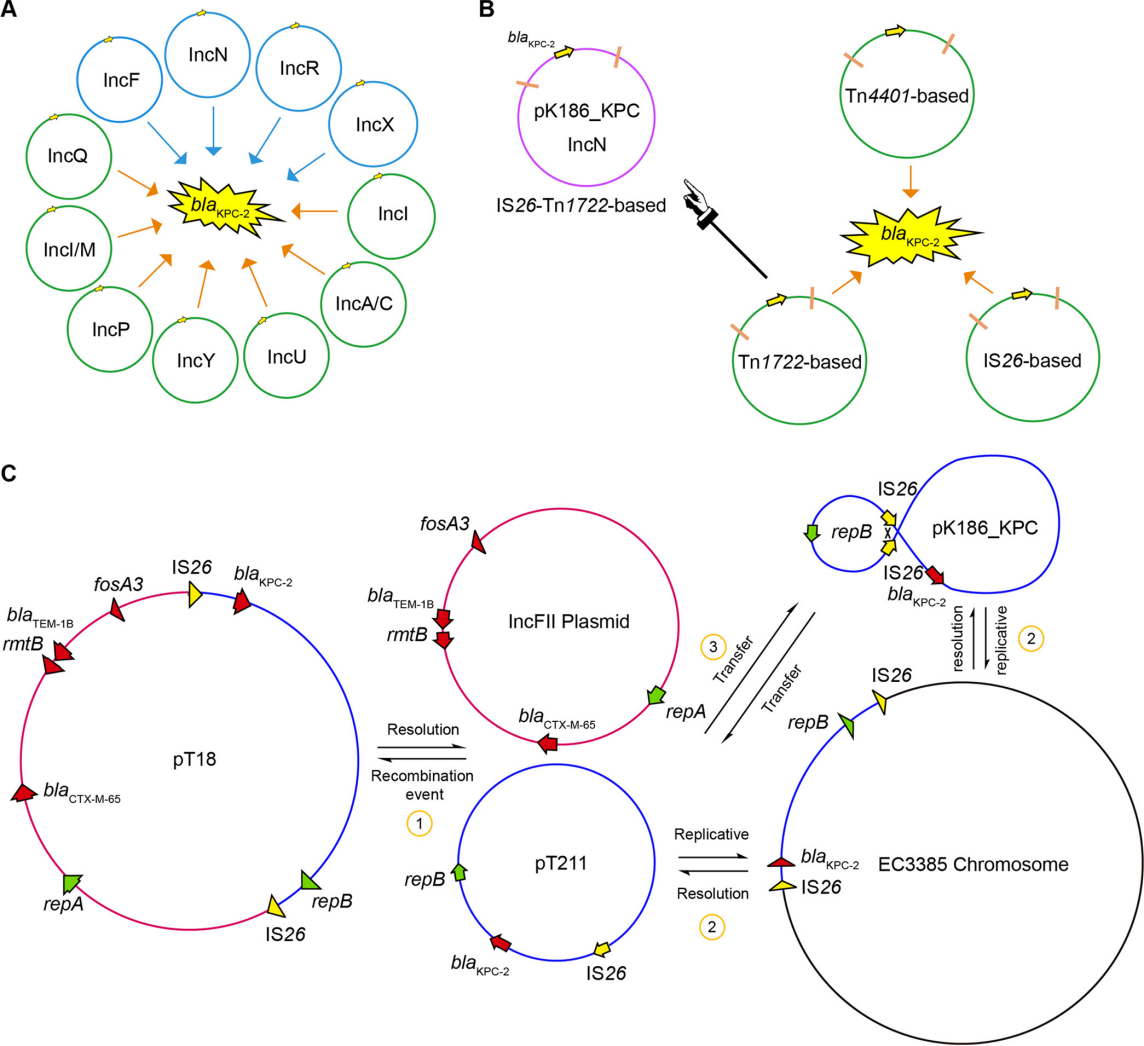
Diversity of K. pneumoniae carbapenemase (KPC-2)-producing plasmids/vehicles and possible patterns for pK186_KPC generation. (A) Scheme for the unexpected complexity in diversified *bla*_KPC-2_-bearing plasmids. To the best of our knowledge, KPC-2 can be tracked to 11 plasmid types. Among these, four types are prevalent, namely, IncN, IncF, IncR and IncX. It is notable that the small, high-copy IncQ subtype (~8.3 kb) of *bla*_KPC-2_-containing plasmids was detected in an international ST15 clone of K. pneumoniae in Brazil in 2015 (21). (B) Genetic environment of plasmid-borne *bla*_KPC−2._ Three types of genetic context separately associate with Tn4401-, Tn1722-, and IS*26*-based composite transposons. Of note, pK186_KPC arises from an IS*26*-Tn1722-based element. (C) The 3-step route is hypothesized to be associated with formation of the ~26-kb, *bla*_KPC-2_-positive translocation unit. (i) The IncN-type plasmid pT211 of P. mirabilis (20) cointegrates with the IncFII plasmid to produce the plasmid pT18 via a certain recombination event, and *vice versa*. (ii) The *bla*_KPC-2_-positive translocation unit occurs on the chromosome of E. coli EC3385. Chromosome-encoded *bla*_KPC-2_ can be enabled by an insertion sequence, IS*26*, to enter into the two plasmids (pT211 and pK186_KPC) (20). (iii) As for pT211 and pK186_KPC, an IS*26*-aided intramolecular replicative transposition probably causes a certain inversion event between the original IS*26* element and the position targeted.

To the best of our knowledge, no less than 11 types of *bla*_KPC-2_-bearing plasmid carrier have been recorded (Fig. 4A) (21). Compared to known plasmids of various sizes (~60 to ~230 kb), the discovery of pK186_KPC supplemented an additional example of s *bla*_KPC-2_-positive plasmid of ~26 kb. Among these, the four most prevalent types include IncR, IncF, IncN, and IncX (Fig. 4A). In total, three kinds of genetic contexts were assigned to the plasmid-borne *bla*_KPC-2_ resistance determinant. Namely, they indicated (i) Tn*4401*-like transposons with IS*Kpn7*-*bla*_KPC-2_-IS*Kpn6* as their core structure (exemplified with pKPC-NY79); (ii) Tn*1722*-based transposons featuring a core structure of IS*Kpn27*-*bla*_KPC-2_-ΔIS*Kpn6* (e.g., pKP048, p628-KPC, pKPC-LK30, etc.) and (iii) an “IS*Kpn27*-*bla*_KPC-2_-ΔIS*Kpn6*” cassette-centering, IS*26*-like transposal element (represented by pECN580) (Fig. 4B). As for pK186_KPC, it appeared as an IS*26*-Tn*1722*-based mini-version, probably arising from a certain Tn*1722*-based transposon with an adjacent IS*26* element.

### Possible pattern of pK186_KPC generation.

To test the conjugative ability of pK186_KPC, a clinical isolate of K186 was mated with the laboratory strain E. coli J53 in LB broth. We failed to recover any transconjugants carrying pK186_KPC after three rounds of independent mating trials. Combined with the plasmid sequence, this finding indicated that pK186_KPC might be a nonconjugative plasmid. However, it cannot rule out the possibility that pK186_KPC can be transferred at an extremely low frequency along with the helper plasmid pK186_1 encoding T4SS and relaxase (Table 2). Presumably, the *bla*_KPC-2_-containing region (~26 kb) is horizontally transferred among the P. mirabilis plasmid, K. pneumoniae plasmid, and E. coli chromosome (Fig. 4C). In brief, the genetic events can be described as follows: (i) an initial transfer of the *bla*_KPC-2_ gene to the IncN-type plasmid via the transposon Tn*1722*, and (ii) an *IS*26-based spreading of a *bla*_KPC-2_ gene-including mobile element. It seems most likely that K. pneumoniae can act as an intermediate host to transmit *bla*_KPC-2_ to P. mirabilis and E. coli, which implies a risk of cross-species transmission (Fig. 4C).

### The lack of fitness cost caused by pK186_KPC.

**FIG 5:**
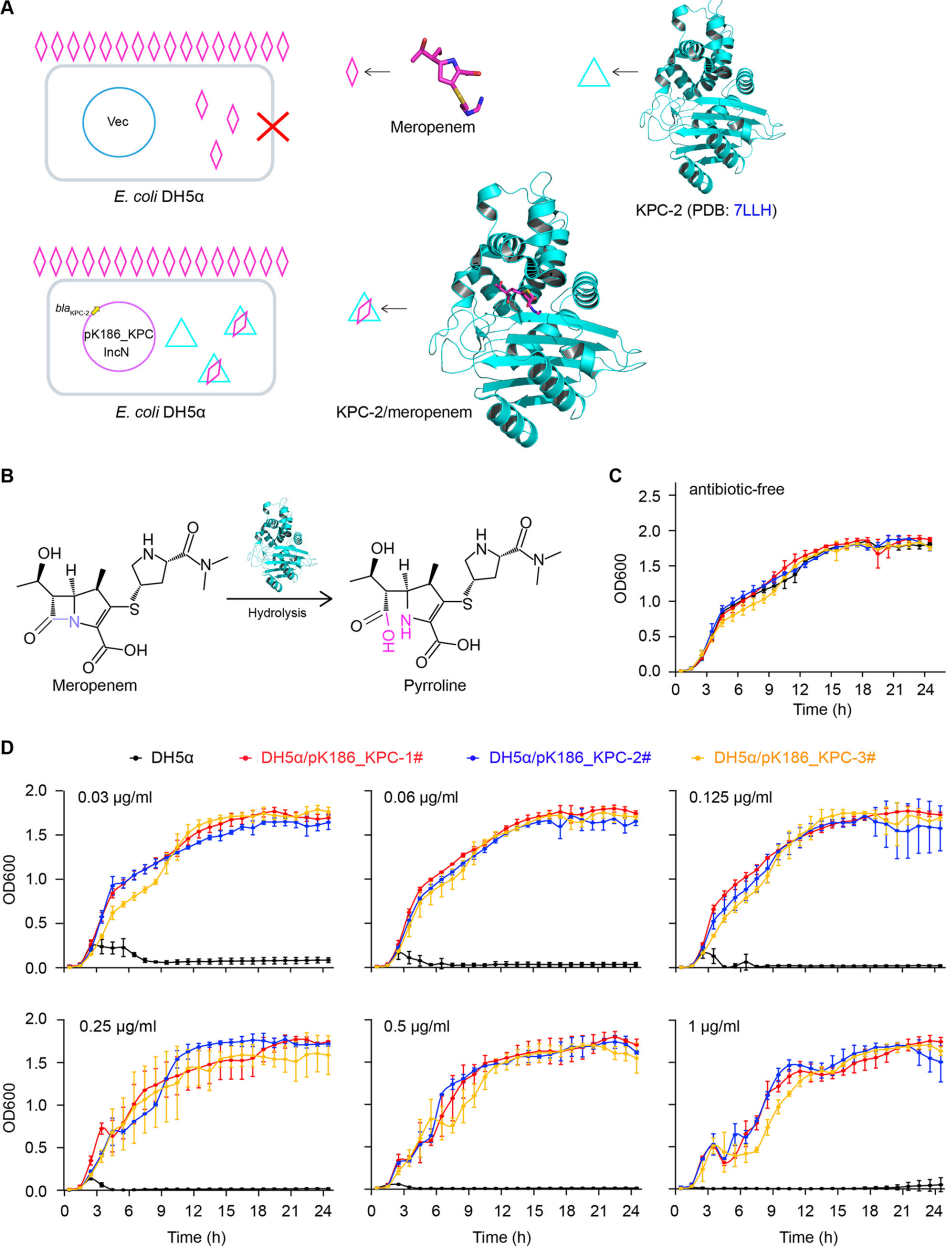
Mechanism of KPC-2 action in E. coli. (A) Schematic representations of meropenem killing and KPC-2 meropenem resistance. The introduction of pK186_KPC can render the recipient E. coli DH5α insensitive to meropenem. In panel A, pink diamonds indicate meropenems and cyan triangles denote KPC-2 protein. The ribbon structure of KPC-2 is shown (PDB accession no. 7LLH). (B) Chemical reaction of meropenem inactivation by KPC-2. (C) Under the antibiotic-free growth condition, no effect on the growth of the E. coli DH5α strains was observed, regardless of *bla*_KPC-2_. (D) Analyses of growth curves suggested no evidence for a fitness cost caused by KPC-2 in E. coli. Three different colonies of DH5α carrying pK186_KPC were tested, designated DH5α/pK186_KPC-1, DH5α/pK186_KPC-2, and DH5α/pK186_KPC-3, respectively. Growth curves were plotted using data from three independent experiments, shown as means ± standard deviation (SD). The recipient control strain, E. coli DH5α, is colored black. Three individual transconjugants are colored separately, with red for DH5α/pK186_KPC-1, blue for DH5α/pK186_KPC-2, and orange for DH5α/pK186_KPC-3. The levels of meropenem supplemented into the culture are shown in panel D (0.03, 0.06, 0.125, 0.25, 0.5, and 1 μg/mL).

As we know, the principle by which carbapenems (e.g., imipenem and meropenem) kill bacterial species relies on their ability to interfere with the synthesis of bacterial cell wall (Fig. 5A). Expression of KPC-2-type β-lactamase efficiently hydrolyzes carbapenem-class antibiotics, rendering the recipient E. coli insensitive to meropenem (Fig. 5A and B). To determine whether or not pK186_KPC results in a fitness cost, we engineered several strains of E. coli DH5α. First, under the meropenem-free condition, the growth curves of E. coli DH5α strains were almost identical, regardless of pK186_KPC (Fig. 5C). Second, the presence of pK186_KPC failed to exert retardation on E. coli growth under an array of growth conditions at various meropenem levels (0.03 μg/mL to 1.0 μg/mL) (Fig. 5C). Consistent with the results for pT211 (20), pK186_KPC seemed to not cause a fitness cost in E. coli DH5α.

### Existence of two companion plasmids pK186_1 and pK186_2.

**FIG 6:**
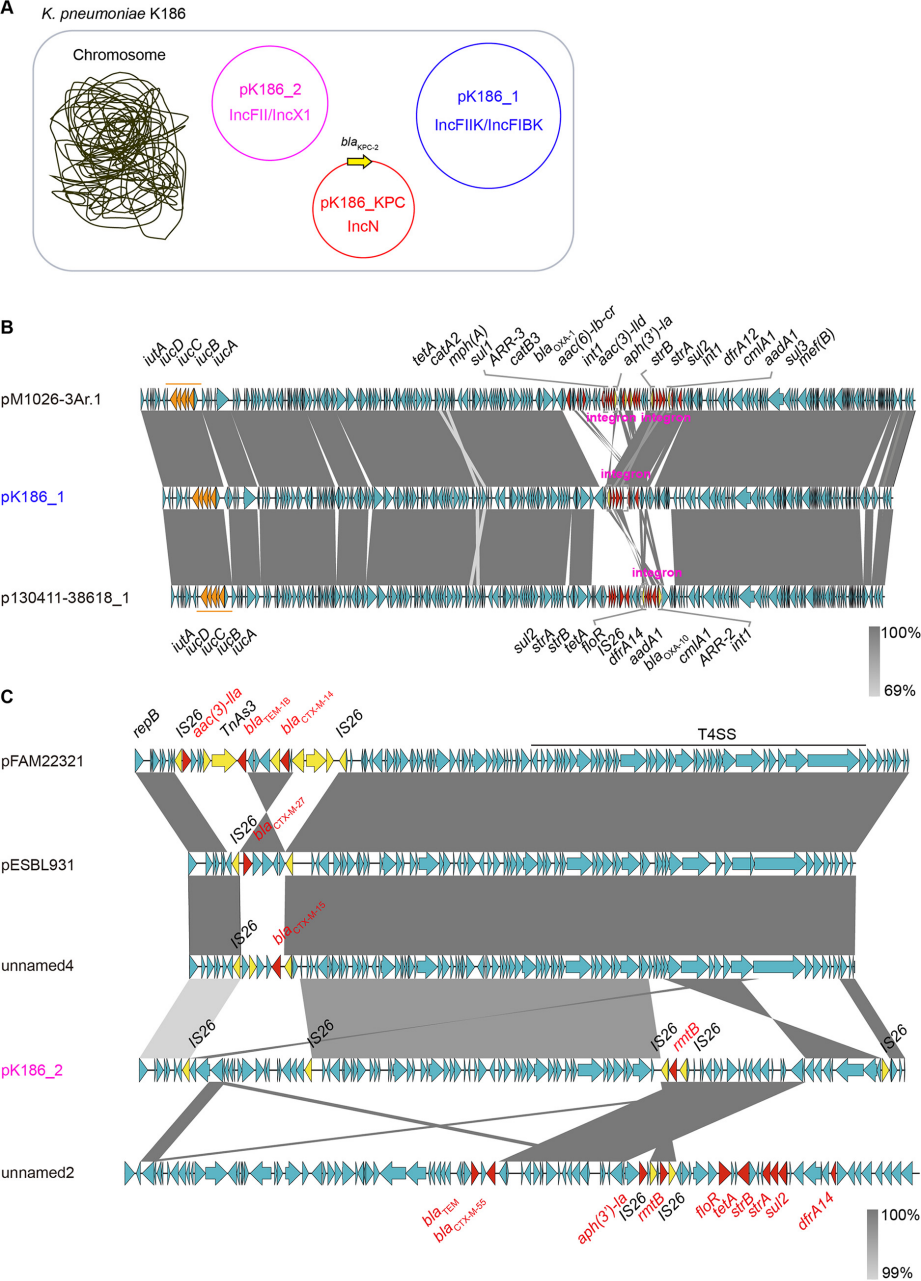
Genomic analysis of the two additional resistance plasmids carried by K. pneumoniae K186. (A) Scheme for a unique carbapenem-resistant strain of K. pneumoniae, K186, which carries three distinct plasmids. In addition to the IncN-type plasmid, pK186_KPC, the remaining two plasmids are pK186_1 and pK186_2. The IncFIIK/IncIBK-type plasmid pK186_1 contains a number of antibiotic resistance genes (namely, *dfrA12*, *sul3*, *aph(3′)-la*, *aadA1*, *aadA2*, *mef(B)*, and *cmlA1)* and virulence genes (*iucABCD* and *iutA*). The IncFII/IncX1-type plasmid pK186_2 encodes *rmtB*, the resistance determinant. (B) Co-linear alignment of pK186_2 with two closely related plasmids, p130411-38618_1 and pM1026-3Ar.1. Apart from a shared backbone, all three plasmids contain the aerobactin region (i.e., *iucABCD-iutA* operon), along with an integron of variable size. (C) Sequence comparison of pK186_2 with four closely related plasmids. The four relative sequences consist of two plasmids (pFAM22321 [accession no. KU288634] plus pESBL931 [CP016389]) and two contigs [unnamed4 (accession no. CP033629) and unnamed2 (CP034761)]. Red arrows denote antimicrobial resistance (AMR) genes, yellow arrows indicate mobile elements, and orange arrows indicate an aerobactin gene cluster. Regions of >99% similarity are shaded dark. pK186_2 appears as an IncFII/IncX1-type hybrid plasmid. This is because (i) it possesses an IncX1 plasmid backbone identical to that of unnamed2 and (ii) shares an IncFII backbone with the three cousins (pFAM22321, pESBL931, and unnamed4).

Apart from pK186_KPC, two companion plasmids designated pK186_1 and pK186_2 also occurred in the clinical strain K186 of ST437 K. pneumoniae (Fig. 3A and 6A). The first, pK186_1, acts as an IncFII_K_/IncFIB_K_-type hybrid plasmid, of which the plasmid backbone and aerobactin region seemed to be shared with two reference plasmids of pM1026-3Ar.1 (accession no. CP063859) and p130411-38618_1 (MK649826) (Fig. 6B). Notably, these three plasmids differed in certain integration-associated regions which feature various AMR loci presumably introduced by distinct integron cassettes. These included (i) *dfrA12* (sulfonamide), (ii) *sul3* (sulfonamide), (iii) *aph(3′)-la* (kanamycin), (iv) *aadA1* (aminoglycoside), (v) *aadA2* (aminoglycoside), (vi) *mef(B)* (macrolide) and (vii) *cmlA1* (chloramphenicol) (Table 2). This agreed with the phenotypic resistance of K186 to numerous antibiotics (Table 1). Along with *iutA*, which encodes an outer membrane ferric aerobactin receptor, pK186_1 also harbors an aerobactin cluster, *iucABCD*. The type of aerobactin encoded by pK186_1 is *iuc3*, instead of the *iuc1* we recently observed in K. pneumoniae isolates from the same hospital (11). Notably, the K186 strain lacks *rmpA*, which encodes a regulator of capsule polysaccharide synthesis. Probably, the absence of putative virulence determinants is partially, if not entirely, relevant to the reduced pathogenesis of the K186 strain (Fig. 2).

As the PlasmidFinder-based analysis stated, pK186_2 belongs to an IncFII/IncX1-type plasmid. Subsequent sequence alignment showed that (i) the IncFII plasmid backbone is analogous to those of pFAM22321 (accession no. KU288634), pESBL931 (CP016389), and unnamed4 (CP033629), and that (ii) pK186_2 shares an IncX1 plasmid backbone with unnamed2 (CP034761) (Fig. 3C). Not surprisingly, *rmtB*, which confers resistance to aminoglycosides, is located between the IS*26* sites of pK186_2 (Fig. 6C). Obviously, the presence of AMR in the two distinct plasmids (pK186_1 and pK186_2) explains in part (if not entirely) the phenotypic resistance of clinical isolate K186 (Table 1).

## CONCLUSION

The global spread of carbapenem-resistant Enterobacterales (CRE) constitutes a substantial threat to health care worldwide, as it renders the carbapenem spectrum (including imipenem and meropenem), an ultimate line of defense antibiotics, clinically useless for antibacterial treatment (22, 23). The China Antimicrobial Surveillance Network (CHINET) allowed us to recognize that the current CRE epidemic situation is devastating, as the positive ratio of meropenem resistance in CRKP detection increases annually, from 2.9% in 2005 to 27.1% in 2021 (http://www.chinets.com/Data/GermYear) (23–25). It seems likely that Zhejiang is the third province in China (in addition to the Henan and Jiangxi provinces) with a CRKP detection rate of over 35%, as of 2020 (http://www.chinets.com/Chinet). This is why intensive investigations have been conducted to closely monitor the transmission dynamics of CRKP (14, 22, 24). In principle, carbapenem destruction by the KPC-2 enzyme is the leading biochemical machinery which accounts for the ongoing prevalence of CRKP. Retrospectively, CRKP initially appeared abroad in 1997 (26), and the first Chinese isolate of *bla*_KPC-2_-harboring K. pneumoniae emerged in Zhejiang Province in 2007 (27). Most *bla*_KPC-2_ is detected in sequence type ST11 of K. pneumoniae, certain types of which contain a certain pLVPK-like virulence plasmid (8, 11, 28). It was noted that stool samples of inpatients only function as colonizer reservoirs for ST11 CRKP (29). Moreover, KPC-2-producing plasmids differ markedly in the following aspects: plasmid size, replication type, and genetic context, highlighting diversified vehicles of carbapenem resistance (11, 13, 30). Certain mutations of *bla*_KPC-2_ which accordingly generate new KPC variants (like *bla*_KPC-31_ [31], *bla*_KPC-51_, and *bla*_KPC-52_ [32]) extend bacterial resistance to cefiderocol and ceftazidime-avibactam, two newly commercialized β-lactam antibiotics. Thus, it is necessary to further track the microevolution of *bla*_KPC-2_ in ST11 CRKP in a One Health context consisted of environmental, animal, and human sectors (33).

Here, we describe K186, a carbapenem-resistant strain of K. pneumoniae containing three distinct plasmids, namely, pK186_KPC, pK186_1, and pK186_2 (Fig. 2A). Both pK186_1 and pK186_2 are hybrid plasmids, rendering K186 insensitive to a spectrum of antimicrobials (such as amikacin and gentamicin). Unlike the prevalent combination of *bla*_KPC-2_-harboring ST11 CRKP in Zhejiang Province, the data reported here provide additional examples of rare ST437 CRKP with KPC-2 production. This is unusual, but not unprecedented. We noticed that carriage of *bla*_KPC-2_ by ST437 of K. pneumoniae was originally detected in a Brazilian hospital in 2011 (34) and then detected in urban rivers in Brazil in 2014 (35). Worryingly, this high-risk clone of ST437 K. pneumoniae producing KPC-2 was found to have acquired the most prevalent determinant of mobile colistin resistance, *mcr-1*, in Brazil in 2018 (36). This potentially compromises the clinical use of both carbapenems and polymyxin as ‘last-resort’ defense options (37, 38). Recently, a Chinese isolate of ST437 CRKP was sampled in which carbapenemase was produced by *bla*_OXA-232_ instead of *bla*_KPC-2_ (39). Unlike the dominant version of large KPC-2-producing vectors (11, 24), *bla*_KPC-2_ is carried by IncN-type pK186_KPC, a small plasmid measuring around 26 kb in our case (Fig. 3B). In fact, an even smaller version of pK186_KPC, pT211, measuring ~24.2 kb, was isolated from Proteus mirabilis from human sputum in Zhejiang Province in 2013, raising the possibility of cointegration of KPC-2-expressing vehicles (20). Compared to pT211 of P. mirabilis, pK186_KPC of K. pneumoniae, collected in Zhejiang in 2017, has an extra IS*26* insertion sequence, indicating a relic of evolutionary events (Fig. 6C). This finding augments the possibility that cross-species transmission of IncN plasmid-borne *bla*_KPC-2_ occurs (but is not limited to) between P. mirabilis and K. pneumoniae. However, we are not aware of an ancestral bacterial host for this rare type of KPC-2-expressing carrier. Collectively, this study, along with recent observations by Li et al. (11), constitutes an ongoing arsenal of diversified vehicles facilitating transferability of KPC-2 carbapenem resistance.

## MATERIALS AND METHODS

### Bacterial strains and media.

The Klebsiella pneumoniae isolate K186 was collected from a patient (Patient A) who was admitted to the Second Affiliated Hospital of Zhejiang University in 2017. As described in the methods of Li et al. (11), we identified it as K. pneumoniae using both matrix-assisted laser desorption ionization–time of flight mass spectrometry and 16S rRNA gene sequencing. To evaluate its potential fitness cost, the plasmid pK186_KPC was electroporated into E. coli DH5α. Three kinds of medium were used to cultivate K186 and E. coli J53 (DH5α or ATCC 25922) at 37°C. These included (i) MacConkey agar medium, (ii) Luria-Bertani broth (LB) agar plates, and (iii) LB liquid medium. When necessary, antibiotics were supplemented accordingly.

### Antimicrobial susceptibility testing.

To determine the resistance profile of K186, routine antimicrobial susceptibility tests were performed for 14 different antibiotics. Following the general guidance of CLSI methods and interpretations, MICs were assigned to the K186 strain. Of note, tigecycline susceptibility was interpreted in accordance with the relevant FDA criteria. The E. coli ATCC 25922 strain acted as a quality control in all MIC experiments.

### Conjugation assays.

The transferability of the pK186_KPC plasmid was assessed using conjugation experiments. Strain K186 acted as a donor, and sodium azide-resistant E. coli J53 functioned as a recipient. In principle, E. coli J53 transconjugants were selected on LB agar plates containing sodium azide (100 μg/mL), and meropenem (0.5 μg/mL) was supplemented for pK186_KPC conjugation. As for possible transconjugants, both multiplex PCR and Sanger sequencing were conducted to verify the presence of pK186_KPC (40). The identity of each transconjugant was confirmed with 16S rRNA gene sequencing. The conjugation frequency was calculated accordingly.

### Infection of *Galleria mellonella*.

The wax moth larvae infection model (Galleria mellonella) was routinely used to judge the pathogenicity of K186 as recently described (11), with little modification. Groups of G. mellonella larvae (~300 mg each, Tianjin Huiyude Biotech Company, Tianjin, China) were formed (10 larvae/group). Larvae were challenged with K186 (in log-phase culture) at 10^6^ CFU and recorded for 72 h postinfection. The larvae infection experiments were conducted in triplicate.

### String test.

The mucoviscosity of K186 was examined using the string test. K186 was inoculated on Columbia blood (5%) agar plates (Oxoid, Thermo Fisher Scientific, Waltham, MA) and kept at 37°C overnight. A toothpick which touched a single colony produced a string when pulled upwards. The cutoff criterion (positive result for the string test) was a viscous string of >5 mm in length.

### S1-PFGE and Southern blotting.

Southern blotting combined with S1-pulsed field gel electrophoresis was performed to determine the physical size of the *bla*_KPC-2_-bearing plasmid pK186_KPC. Southern blotting was conducted in accordance with the manufacturer’s protocol. First, the DIG-labeled *bla*_KPC-2_ probe was prepared using a DIG High Prime DNA Labeling and Detection kit (Roche AG, Basel, Switzerland). Second, chromosomal (and/or plasmid) DNA was transferred onto a positively charged nylon membrane, cross-linked using a microwave oven, and hybridized with the *bla*_KPC-2_-specific probe. As for S1-PFGE, the CHEF Mapper XA system (Bio-Rad Laboratories, Hercules, CA) was used and *S1* nuclease (TaKaRa Bio) was utilized.

### Genome sequencing, assembly, and bioinformatic analysis.

Genome DNA was extracted from overnight culture and subjected to whole-genome sequencing using both the HiSeq platform (Illumina, San Diego, CA) and a Nanopore PromethION (Oxford Nanopore Technologies, Oxford, United Kingdom). Illumina and Nanopore sequencing reads were hybrid-assembled using Unicycler v0.3.0. Plasmids sequences were annotated using RAST v2.0 along with BLASTp/BLASTn searches (rapid annotation using subsystem technology [http://rast.nmpdr.org]). The sequences of the K186 chromosome and plasmids (namely, pK186_1, pK186_2, and pK186_KPC) were deposited in GenBank under four accession numbers, CP076518 to CP076521.

Plasmid incompatibility typing was determined using PlasmidFinder v1.3 (https://cge.cbs.dtu.dk/services/PlasmidFinder-1.3/). Antibiotic resistance determinants were examined using ResFinder v3.1 (https://cge.cbs.dtu.dk/services/ResFinder/). Chromosomal multilocus sequence typing (MLST), virulence loci (*ybt*, *iro*, *iuc*, *rmpA*, and *rmpA2*), and K and O antigen loci were typed using Kleborate v0·3·0 (https://github.com/katholt/Kleborate). ISfinder v2.0 (https://www-is.biotoul.fr/index.php) was used to determine insertion sequences. Easyfig v2.2.2 was used for multiple alignments of genomic loci. oriTfinder allowed us to detect the origins of transfer sites (42).

### Growth curves.

To evaluate potential fitness costs, bacterial growth curves were determined. In brief, log-phase cultures (OD_600_ [optical density at 600 nm] adjusted to 0.5) were inoculated (1:1,000; vol/vol) in a 96-well glass-bottomed plate (supplemented with LB liquid medium, 200 μL/well). Subsequently, the plate was kept in a spectrophotometer (Spectrum Lab S32A) set at 37°C and shaken at 300 rpm for 24 h. The OD_600_ was determined at 1-h intervals.

### Data availability.

The chromosome sequences of clinical isolate K186 of K. pneumoniae were deposited under the accession number CP076518. The three companion plasmids of K186 were pK186_1, pK186_2, and pK186_KPC, respectively. Plasmid sequences were deposited under the following accession numbers: CP076519 for pK186_1, CP076520 for pK186_2, and CP076521 for pK186_KPC.
