# Supplementary material for: A Small KPC-2-Producing Plasmid in Klebsiella pneumoniae: Implications for Diversified Vehicles of Carbapenem Resistance
Source: Microbiol Spectr. 2022 May 17;10(3):e02688-21. doi: 10.1128/spectrum.02688-21 (PMC9241637; doi:10.1128/spectrum.02688-21)
Supplement: SUPPLEMENTAL FILE 1 — Supplemental material. Download spectrum.02688-21-s001.pdf, PDF file, 0.1 MB [file spectrum.02688-21-s001.pdf]

Supplemental Information

Supplemental Tables

Table S1 Clean reads of K186 strain

Sample ID	Seq Num	Sum Base (bp)	N50 Len (bp)	N90 Len (bp)	Mean Len (bp)	Max Len (bp)	Mean Qual
K186	91,412	1,436,722,127	24,641	7,587	15,716	150,245	10.17
